# DEVELOPMENT OF A NOVEL PHENO FRAILTY INDEX FOR MICE

**DOI:** 10.1093/geroni/igae098.2755

**Published:** 2024-12-31

**Authors:** Brady Samuelson, Dantong Zhu, Alice Kane

**Affiliations:** Institute for Systems Biology, Seattle, Washington, United States; Institute for Systems Biology, Seattle, Washington, United States; Institute for Systems Biology, Seattle, Washington, United States

## Abstract

Frailty is assessed in both model organisms and people with frailty index tools that quantify health related deficits. Here we aim to develop a new mouse frailty assessment tool (Pheno-FI) based on clinically-relevant physiological and functional measures that are easily obtainable, and can be measured longitudinally. The Pheno-FI score was developed using male and female C57BL/6 mice (N=40) at 12 months of age. We selected 17 items to include that changed with age, were health-associated, and were not correlated with each other. These included variables like HR, PLT, RBC, WBC, basophils, QRS, QT, open field, glucose, nesting and burrowing to provide a holistic view of frailty. The standard deviation (±1.5) of the baseline mice were used to determine the sex-specific cut points for our deficits, and if values were outside these cut points they were given a score = 1, and within the cut points a 0. Pheno-FI is the average of these items. Once developed in the baseline data, the Pheno-FI was calculated for male and female mice aged 12 to 36 months. Pheno-FI scores increase with age in both males and females, and are positively correlated with the previously validated mouse clinical frailty index (Females R2=0.6, p= 7.7e8 Males R2=0.62, p= 3.1e11). Additionally, Pheno-FI scores show a negative correlation with lifespan (Female R2= -0.56 p=6.7e-7 Male R2= -0.5 p=2.3e7). The Pheno-FI provides a new tool for the field to classify frailty in longitudinal aging mouse studies, to explore frailty mechanisms, biomarkers and interventions.

